# Acyl-CoA synthetase 6 regulates long-chain polyunsaturated fatty acid composition of membrane phospholipids in spermatids and supports normal spermatogenic processes in mice

**DOI:** 10.1096/fj.201901074R

**Published:** 2019-11-25

**Authors:** Kyosuke Shishikura, Sayoko Kuroha, Shinnosuke Matsueda, Hachiro Iseki, Takeshi Matsui, Azusa Inoue, Makoto Arita

**Affiliations:** * Laboratory for Metabolomics, Riken Center for Integrative Medical Sciences (IMS), Tsurumi, Yokohama, Japan;; † Cellular and Molecular Epigenetics Laboratory, Graduate School of Medical Life Science, Yokohama City University, Tsurumi, Yokohama, Japan;; ‡ Division of Physiological Chemistry and Metabolism, Graduate School of Pharmaceutical Sciences, Keio University, Minato-ku, Japan;; § Laboratory for Skin Homeostasis, Riken Center for Integrative Medical Sciences (IMS), Tsurumi, Yokohama, Japan;; ¶ Laboratory for Metabolic Epigenetics, RIKEN Center for Integrative Medical Sciences (IMS), Tsurumi, Yokohama, Japan

**Keywords:** testis, ACSL6, docosahexaenoic acid, docosapentaenoic acid

## Abstract

Long-chain polyunsaturated fatty acids (LCPUFAs), such as docosahexaenoic acid (DHA, 22:6) and docosapentaenoic acid (DPA, 22:5), have versatile physiologic functions. Studies have suggested that DHA and DPA are beneficial for maintaining sperm quality. However, their mechanisms of action are still unclear because of the poor understanding of DHA/DPA metabolism in the testis. DHA and DPA are mainly stored as LCPUFA-containing phospholipids and support normal spermatogenesis. Long-chain acyl-conenzyme A (CoA) synthetase (ACSL) 6 is an enzyme that preferentially converts LCPUFA into LCPUFA-CoA. Here, we report that ACSL6 knockout (KO) mice display severe male infertility due to attenuated sperm numbers and function. ACSL6 is highly expressed in differentiating spermatids, and ACSL6 KO mice have reduced LCPUFA-containing phospholipids in their spermatids. Delayed sperm release and apoptosis of differentiated spermatids were observed in these mice. The results of this study indicate that ACSL6 contributes to the local accumulation of DHA- and DPA-containing phospholipids in spermatids to support normal spermatogenesis.—Shishikura, K., Kuroha, S., Matsueda, S., Iseki, H., Matsui, T., Inoue, A., Arita, M. Acyl-CoA synthetase 6 regulates long-chain polyunsaturated fatty acid composition of membrane phospholipids in spermatids and supports normal spermatogenic processes in mice.

Long-chain polyunsaturated fatty acids (LCPUFAs) are composed of a 20- or 22-carbon chain length with multiple double bonds (1, 2). LCPUFAs are generally present in the forms of phospholipids and neutral lipids in tissues. The fatty acid composition is different among tissues, which may contribute to normal tissue function and homeostasis (3). However, the physiologic function of LCPUFAs and LCPUFA-containing phospholipids largely remains elusive because of the limited understanding of the molecular mechanisms underlying LCPUFA metabolism in LCPUFA-enriched cells and tissues.

Once taken up into the cytoplasm, free fatty acids are activated to form acyl-coenzyme A (CoA) and are eventually incorporated into phospholipids or neutral lipids. In the testis, docosahexaenoic acid (DHA, 22:6 n-3) and docosapentaenoic acid (DPA, 22:5 n-6) are highly enriched (3, 4). Lysophospholipid acyltransferase 3 (LPAAT3) is abundantly expressed in spermatids and selectively incorporates DHA into phospholipids in the testis (5). LPAAT3 knockout (KO) mice display severe male infertility with abnormal sperm morphology (5). Long-chain acyl-CoA synthetase (ACSL) is an enzyme that synthesizes long-chain acyl-CoA from long-chain fatty acids. ACSLs consist of 5 isoforms (ACSL1 and 3–6); each isoform has a different substrate preference (6). Among the isoforms, ACSL6 preferentially incorporates DHA and related LCPUFAs into phospholipids *in vitro* (7, 8). Of note, the tissue distribution of ACSL6 gene expression largely overlaps with the relative abundance of DHA-containing phospholipids (9). Recently, Chouinard *et al.* (10) reported that ACSL6 is involved in the selective incorporation of DHA into brain phospholipids. Here, we report for the first time that ACSL6 is also involved in the selective enrichment of DHA- and DPA-containing membrane phospholipids in differentiating spermatids, which supports normal spermatogenesis in mice.

## MATERIALS AND METHODS

### Generation of ACSL6 KO mouse strain

**Figure 1:**
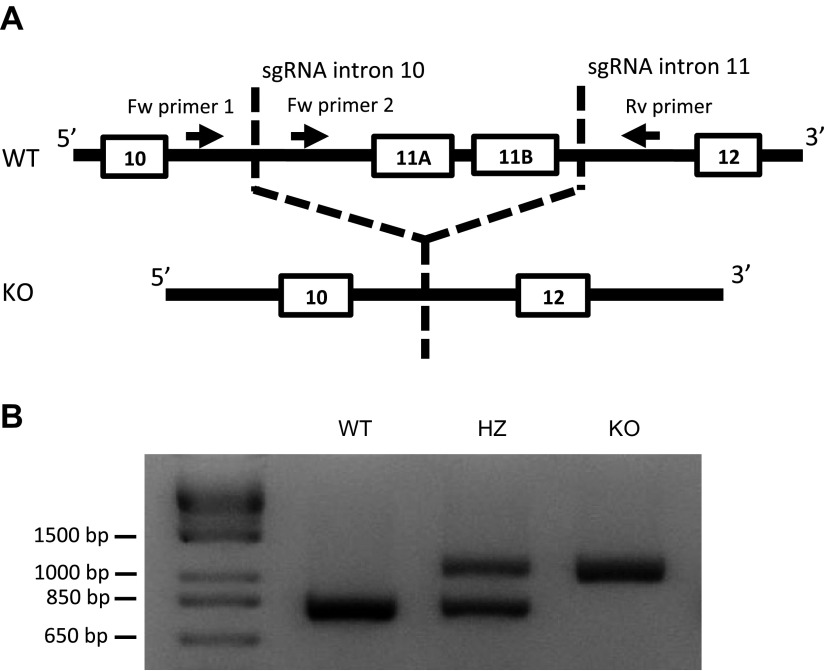
Construction of ACSL6 KO mice. *A*) ACSL6 KO mice were produced by CRISPR/Cas9 using sgRNAs targeting introns 10 and 11. A 1-kbp-deletion mutant was produced that lacked 2 copies of exon 11. Using 2 forward (Fw) primers (located 3′ and 5′ of the deletion site in intron 10) and a reverse (Rv) primer (located 3′ of the deletion site in intron 11), we confirmed the deletion of ACSL6 exon 11. *B*) Genotyping results for ACSL6 WT, HZ, and KO mice. PCR of the WT allele yielded a PCR product from Fw primer 2. PCR of the KO allele amplified a product from Fw primer 1.

The ACSL6 KO mouse strain was generated using the clustered regularly interspaced short palindromic repeats/CRISPR-associated protein-9 nuclease (CRISPR/Cas9) system. Mouse ACSL6 has 2 gate domains, which are coded by exon 11 of *ACSL6* gene and are essential for its catalytic activity (9, 11). We designed 2 single guide RNAs (sgRNAs) that have a common scaffold sequence to delete exon 11 (**Fig. 1*A***). The 2 sgRNAs targeted introns 10 and 11. Cas9 mRNA and 2 sgRNAs were injected into the cytoplasm of C57BL/6J embryos (12). The ACSL6 intron 10 guide sequence was UGUUAGGUCGCAGCGCCAGU(GGG). The ACSL6 intron 11 guide sequence was AGGGAAUCCAUAUAGGACGG(UGG). The scaffold sequence was GUUUUAGAGCUAGAAAUAGCAAGUUAAAAUAAGGCUAGUCCGUUAUCAACUUGAAAAAGUGGCACCGAGUCGGUGCUUU. The protospacer adjacent motif is shown in parenthesis. Genotyping of mice was performed using PCR primer pairs for the wild-type (WT) and deleted sequence of the ACSL6 gene (Fig. 1*B*). Forward primer 1 was 5′-GCTGGGGTTTAATTTGGCCG-3′. Forward primer 2 was 5′-TGATCCAGGTAAGCCTCCGA-3′. The reverse primer was 5′-TGCCCAGCTGCGTAAAACTA-3′. PCRs were performed from 1 cycle of 3 min at 94°C, followed by 45 cycles of denaturation for 30 s at 94°C, primer annealing for 30 s at 58°C, and primer extension for 75 s at 72°C. In the KO allele, PCR product between primer 1 and reverse primer was well amplified, whereas in the WT allele, the product from primer 1 was not detected because of the short extension time. All animal experiments were approved by the Animal Care and Use Committee of Riken Yokohama Institute and Yokohama City University.

### Gene expression analysis

Gene expression was measured using real-time quantitative RT-PCR. Total RNA was purified with the RNeasy Mini Kit and DNase I (Qiagen, Hilden, Germany) according to the manufacturer’s instructions. Total RNA (organs: 1000 ng, spermatids: 40–100 ng) was used to synthesize cDNA using Prime Script RT Master Mix (Takara Bio, Kyoto, Japan). Quantitative PCR was performed in duplicate using gene-specific primers with the Step One Plus Real-Time PCR system (Thermo Fisher Scientific, Waltham, MA, USA) and TB Green Premix Ex Taq II (Takara Bio) for detection. The primer sequences used were as follows: ACSL6, forward 5′-AAGCAGTCGGAAGAAGTGGAG-3′ and reverse 5′-GGCATCGTCATAGTAATGGGTAAG-3′; doublesex and Mab-3 related transcription factor 1 (DMRT1), forward 5′-TTCTGGCTTGGTCTCCCTCTC-3′ and reverse 5′-TCACTCGTCCTCATCCTCTTCC-3′; A-kinase anchoring protein 3 (AKAP3), forward 5′-CGCAAAGACCTGGAGAAAAG-3′ and reverse 5′-ACCTTTTCGTTGGTCCACTG-3′; protamine 2 (PRM2), forward 5′-CCACAAGAGGCGTCGGTCAT-3′ and reverse 5′-CCTTGGCTCCAGGCAGAATG-3′; and glyceraldehyde 3-phosphate dehydrogenase (GAPDH), forward 5′-TGACAATGAATACGGCTACAGCA-3′ and reverse 5′-CTCCTGTTATTATGGGGGTCTGG-3′. Relative mRNA levels were calculated using the ΔΔ*C_t_* method and normalized to GAPDH mRNA (13).

### Lipid extraction from tissues

Lipids were extracted from tissues or cells using single-phase extraction (14). The homogenates were mixed with MeOH and incubated for 1 h at room temperature. A half volume of CHCl_3_ was added, and the samples were incubated for 1 h at room temperature. Finally, water was added, and the extracts composed of MeOH:CHCl_3_:H_2_O (2:1:0.2; v/v) were obtained. After extraction, samples were centrifuged at 2000 *g* for 10 min, and the supernatants were collected.

### Organelle separation

Homogenated testes were separated by centrifugation at 1000 *g* for 5 min to remove cell debris. The supernatant was centrifuged at 8000 *g* for 15 min to isolate mitochondria membrane fraction. The supernatant was further centrifuged at 100,000 *g* for 60 min to isolate microsome fraction. Post-8000 and -100,000 *g* pellets were applied to lipidomics analyses. To ensure the successful separation, we determined the presence of organelle marker proteins by immunoblotting. Anti-GM130 antibody (ab169276) and anti-ATP5A antibody (ab14748) were from Abcam (Cambridge, United Kingdom). Polyclonal anti-protein disulphide-isomerase (PDI) antibody (3501) and antilight chain 3B (LC3B) antibody were from Cell Signaling Technology (Danvers, MA, USA) and MilliporeSigma (Burlington, MA, USA), respectively.

### Liquid chromatography–mass spectrometry–based lipidomics

Lipidomics was performed as previously described by Aoyagi *et al*. (15). Briefly, extracted lipids were measured using the Acquity ultra-performance liquid chromatography (LC) (UPLC) system (Waters, Milford, MA, USA) coupled with a quadrupole/time-of-flight mass spectrometry (MS) (TripleTOF 6600; AB Sciex, Framingham, MA, USA). LC separation was performed using a reverse-phase column [Acquity UPLC BEH Peptide C18 (50 × 1.7–mm inner diameter, 2.1-µm particle size; Waters)]. Samples were injected in 1 µl for tissue extracts and 3 µl for cell extracts.

### Quantification of lipids

Detected species were annotated and quantified with MS-DIAL using peak height values (16). To calculate the fatty acid composition of the phospholipids, peak heights of individual species were normalized with the sum of the heights of all the detected species and presented as a percentage of the total phospholipid species (5). Phospholipid species in excess of 1% [phosphatidylcholine (PC), phosphatidylserine (PS), phosphatidylinositol (PI)] or 2% [phosphatidylethanolamine (PE)] of the total species are shown in the figures.

### High-performance thin-layer chromatography and phospholipid measurement

After extraction by the Bligh and Dyer method, total lipids were applied on high-performance thin-layer chromatography plates (MilliporeSigma) using di-palmitate PC, PE, PS, PI, phosphatidylglycerol (PG), and phosphatidic acid (PA) as standards (17). Plates were developed in chloroform:ethanol:water:triethylamine, 30:35:7:35 (v/v/v/v), as previously described by Fuchs *et al*. (18). Each phospholipid *in silica* gel was scraped and extracted with chloroform:methanol, 1:1 (v/v), and the phosphorus content of each fraction was determined after digestion with perchloric acid as previously described by Bartlett *et al*. (19).

### Tissue acyl-CoA synthetase activity

Tissue acyl-CoA synthetase activity was determined as previously described by Narita *et al*. (20) with slight modifications. After collection of the membrane fraction using buffer (0.25 M sucrose, 20 mM Tris-HCl pH 7.5, 1 mM DTT) with protease inhibitor cocktail (Complete EDTA free; MilliporeSigma), protein concentration was determined with bicinchoninic acid assay. The membrane fraction (2 µg protein) was reacted in 100 µl of reaction buffer (200 mM Tris-HCl pH 7.5, 8 mM MgCl_2_, 1 mM EDTA, 1 mM NaF, 1 mg/ml bovine serum albumin, 2.5 mM ATP, 0.5 mM CoA, 2.5 µM 16:0-d31, 2.5 µM 20:4-d5, 2.5 µM 22:5, and 2.5 µM 22:6). The reaction was terminated with methanol. Samples were then dried up and reconstituted in methanol:chloroform:water (2:1:0.2, v/v/v). After centrifugation at 2000 *g* for 10 min, the supernatant was applied to LC-MS.

### Acyl-CoA measurement

Acyl-CoAs were measured with Acquity UPLC system coupled with a triple-quadropole MS (Qtrap 6500; AB Sciex). LC separation was performed using a reverse-phase metal-free column [YMC-triart C18 (50 × 2.1–mm inner diameter, 1.9-µm particle size; YMC, Kyoto, Japan)] with a gradient elution of mobile phase A [methanol:acetonitrile:water (1:1:3, v/v/v) containing 50 mM ammonium acetate, 500 nM EDTA, and 0.025% ammonium hydroxide] and mobile phase B (100% isopropanol containing 50 mM ammonium acetate, 500 nM EDTA, and 0.025% ammonium hydroxide). Column temperature was set at 40°C, and flow rate was 0.25 ml/min. For the separation, gradient elution started at 100% A, and after 1 min of hold time, B was increased linearly to 50% at 4 min and 95% at 6 min. Eluted acyl-CoAs were detected with multiple reaction monitoring (MRM) mode by using neutral loss of 3-phosphate-adenosine-5-diphosphate moiety (21). Acyl-CoAs were quantified by measuring the metabolite peak area as a ratio to the area of internal standard. The internal standard was 17:0 CoA (Avanti, Alabaster, AL, USA).

### Single testicular cell sorting

After harvesting the testis, the tunica albuginea was removed and digested with 1 mg/ml collagenase type I (Wako, Osaka, Japan) in DMEM for 10 min at 37°C. The digested samples were centrifuged at 350 *g* at 20°C for 5 min. The pellets were treated with 0.25% trypsin (Wako) and DNase Type I (Wako) in PBS at 37°C for 10 min. The reaction was neutralized with 10% fetal bovine serum/DMEM. A single-cell suspension was obtained by passage of the cells through a 40-µm cell strainer. Testicular single cells were stained with 2.5 µg/ml Hoechst 33342 (Dojindo Laboratories, Kumamoto, Japan) in 10% fetal bovine serum/DMEM at 33°C for 60 min. After centrifugation at 350 *g* for 5 min at 4°C, cells were stained with 1 µg/ml propidium iodide. Stained cells were analyzed and sorted using the BD fluorescence-activated cell sorting Aria flow cytometer (BD Biosciences, San Jose, CA, USA). To obtain highly purified spermatids, we gated cells using a combination of 2 methods (22, 23). The gating is shown in Supplemental Fig. S2.

### Isolation of sperm from the cauda epididymis

Sperm were isolated from the cauda epididymis by cutting and gentle squeezing and then incubated in human tubal fluid (HTF) medium (Merck GmbH, Darmstadt, Germany) at 37°C for 1 h. After the incubation, the sperm were fixed with 2% paraformaldehyde and counted using a cell counting plate (Watson, San Diego, CA, USA).

### Morphologic analysis of epididymal sperm

Fixed sperm were smeared on glass slides and dried at room temperature for 2 h. The samples were washed with PBS and stained with hematoxylin and eosin (HE). Images were obtained by fluorescence microscopy BZ-X710 (Keyence, Osaka, Japan). Sperm that had a tail and nucleus were counted. Sperm that did not have a sharp craw-like head were defined as abnormal. Cell debris was ruled out during counting.

### Oocyte collection and in vitro fertilization

MII-stage oocytes were collected from 8-wk-old Institute of Cancer (ICR) females superovulated with 7.5 I.U. Pregnant mare serum gonadotropin/human chorionic gonadotropin (PMSG/hCG) (ASKA Animal Health, Tokyo, Japan). Sperm were obtained from the caudal epididymis of adult ACSL6 heterozygous (HZ) or KO males in 200 μl HTF medium supplemented with 10 mg/ml bovine serum albumin (MilliporeSigma). Spermatozoa capacitation was attained by 1 h of incubation in HTF medium. For some experiments, the zona pellucida (ZP) was removed from oocytes by a brief treatment with Acidic Tyrode’s solution (Merck). A constant concentration of sperm was used for *in vitro* fertilization (IVF) (4.5–7.0 × 10^5^/ml for ZP-intact IVF). The fertilization ratio was determined 6 h after IVF by observing pronuclei and the second polar bodies. Zygotes were cultured in potassium simplex optimized medium (KSOM) (Merck) in a humidified atmosphere with 5% CO_2_/95% air at 37.8°C.

### Intracytoplasmic sperm injection

MII-stage oocytes were collected from 8-wk-old B6D2F1/J (BDF1) females superovulated with 7.5 I.U. PMSG and hCG. Cumulus cells were dissociated from the MII oocytes by treatment with M2 medium containing hyaluronidase (Merck), and the oocytes were incubated in KSOM medium. Sperm collection and capacitation were performed in HTF as described above. After cutting the sperm tails in 10% polyvinylpyrrolidone (PVP) solution (Irvine Scientific, Santa Ana, CA, USA) using an impact-driven piezomicromanipulator, sperm heads were injected into MII oocytes. The oocytes were then transferred back to KSOM medium.

### Histologic analysis

The testes were fixed with Davidson’s fixative for 24 h at room temperature and embedded in paraffin. Samples were cut and stained with HE. TUNEL staining was performed using the *In Situ* Cell Death Detection Kit (MilliporeSigma) according to the manufacturer’s instructions. Images were obtained using the Leica TCS SP8 Laser Scanning Confocal Microscope (Leica, Wetzlar, Germany).

### Field emission scanning electron microscopy

Perfusion fixation was performed through the left ventricle with 2.5% glutaraldehyde in 0.1 M phosphate buffer (pH 7.4). The testes or epididymis were then immersed in the same fixative for 2 h at 4°C. After rinsing 3 times with 0.1 M phosphate buffer, the samples were postfixed with 2% osmium tetroxide (Taab, Aldermaston, United Kingdom) for 1 h at room temperature and then dehydrated for 20 min each with 50, 70, 80, 90, and 95% ethanol and 3 changes of 100% ethanol. After dehydration, tissues were incubated with 100% propylene oxide (2 changes for 20 min each) and then treated with 1:1 propylene oxide–Taab Epon 812 (Taab) mixture overnight at room temperature. Tissues were incubated with 1:2 propylene oxide–Taab Epon 812 mixture for 3 h and then transferred to Epon 812 for 4 h at room temperature. The samples were hardened at 60°C for 2 d in a Cryo Dish (Shoei Works, Tokyo, Japan). After trimming, 3 × 4–mm sections were cut horizontally with a histo diamond knife (Diatome, Hatfield, PA, USA) using an Ultracut UCT Microtome (Leica) at 0.15-µm thickness. The sections were placed on slide glasses and then stained with Toluidine blue (Wako). Images were collected using the BZ-X710 (Keyence). The stained sections were further stained with 0.4% uranium acetate and Reynold’s solution. After drying, they were coated with osmium using an HPC-1SW vacuum evaporator (Shinku Device, Mito, Japan). Electron microscopic images were obtained using the SU8220 field emission scanning electron microscope (Hitachi High Technologies Corp., Tokyo, Japan) with a YAG-BSE detector (5 kV).

### Mitochondrial membrane potential

For the mitochondrial membrane potential (MMP) measurement, sperms from epididymis were stained with 2.5 µg/ml Hoechst 33342 for 20 min at 33°C and then incubated for 10 min in 5 µg/µl JC-1 (Thermo Fisher Scientific) with or without 100 µM carbonyl cyanide m-chlorophenyl hydrazone (CCCP; MilliporeSigma). After staining with Ghost dye Red 780 (Tonbo Bioscience, San Diego, CA, USA), cells were analyzed with fluorescence-activated cell sorting Canto II (BD Biosciences) as previously described by Agnihotri *et al*. (24).

### Measurement of reproductive hormones

Serum follicle stimulating hormone (FSH), LH, and testosterone were quantified using ELISA kits according to the manufacturers’ instructions: Testosterone Parameter Assay Kit KGE010 (R&D Systems, Minneapolis, MN, USA); LH ELISA Kit CEA441Mu (Cloud-Clone, Houston, TX, USA); FSH ELISA Kit CEA830Mu (Cloud-Clone).

## RESULTS

### Male infertility in ACSL6 KO mice

The ACSL6 KO mouse strain was established using the CRISPR/Cas9 system (Fig. 1). The crossing of ACSL6 HZ mice yielded ACSL6 WT, HZ, and KO mice almost at a Mendelian ratio (79:157:65). Although each genotype grew normally, we found that mature ACSL6 KO males (8 wk of age) were completely infertile and failed to produce offspring when mated to 8-wk-old WT females for 12 wk (**Table 1**).

**TABLE 1:** Male infertility of ACSL6 KO mice

Pairs (male × female)	Litter	Litter size
WT × WT (*n* = 9)	23	129
HZ × WT (*n* = 9)	23	142
KO × WT (*n* = 10)	0	0

### Decreased levels of DHA and DPA phospholipids in spermatids

**Figure 2:**
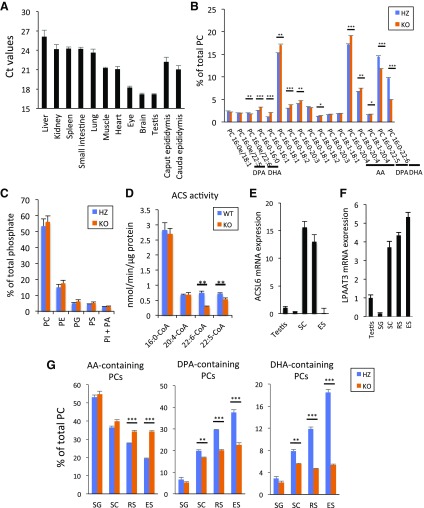
ACSL6 produces DHA phospholipid in spermatids. *A*) *ACSL6* expression was determined by RT-PCR from total RNA extracted from different tissues. *B*) Percentage of total PC in ACSL6 HZ and KO testes. Only phospholipids present in excess of 1% are shown. *C*) Abundance of major phospholipid species in ACSL6 HZ and KO testes. Data are presented as means ± sem (*n* = 3). *D*) Enzymatic activity of WT and KO testes. Data are presented as means ± sem (*n* = 5). *E*, *F*) mRNA expression of *ACSL6* (*E*) and *LPAAT3* (*F*) in isolated spermatids compared with that of the whole testis. mRNA levels were normalized to *GAPDH*. *G*) Percentage of AA (20:4)-, DHA (22:6)-, and DPA (22:5)-containing PC in isolated spermatids. SG, spermatogonia. Data are presented as means ± sem (*n* = 3). *P < 0.05, **P < 0.01, ***P < 0.001 (unpaired Student’s *t* test).

ACSL6 is abundantly expressed in brain, testis, eyes, heart, and skeletal muscle (**Fig. 2*A***). As reported previously (25, 26), DHA- and DPA-containing phospholipids are abundant in the testis. We found that these phospholipid species were significantly reduced in ACSL6 KO mice. In contrast, arachidonic acid (AA, 20:4)-containing PC was significantly increased in ACSL6 KO mice without changing the amount of each phospholipid species (Fig. 2*B, C*). The total acyl-CoA synthetase activity was significantly reduced by 60% for DHA and by 25% for DPA, whereas no change was observed for palmitate and AA in ACSL6 KO testis (Fig. 2*D*). These results indicate that ACSL6 is involved in the selective incorporation of DHA and DPA into the phospholipids of the testis *via* the DHA- and DPA-CoA production (Supplemental Fig. S1). Lipidomic analysis revealed that the changes in fatty acid species in phospholipids were similar among different membrane fractions (Supplemental Fig. S2). To determine the ACSL6 localization in the testes, we isolated different stages of germ cells from the adult mouse testis using flow cytometry (Supplemental Fig. S3). The cell fractions contained diploid spermatogonia, tetraploid spermatocyte (SC), haploid round spermatid (RS), and elongated spermatid (ES) (27). Compared with the average testicular ACSL6 gene expression, SC and RS showed higher ACSL6 mRNA expression (Fig. 2*E*). This gene expression pattern was similar to that of LPAAT3 expression in SC, RS, and ES (Fig. 2*F*). DPA- and DHA-containing PCs were significantly increased during spermatid differentiation, whereas the levels of AA-containing PCs were reduced. Differentiating spermatids from ACSL6 KO mice had reduced levels of DPA- and DHA-containing PCs compared with HZ mice (Fig. 2*G*).

### Decreased sperm number and function in ACSL6 KO mice

**Figure 3:**
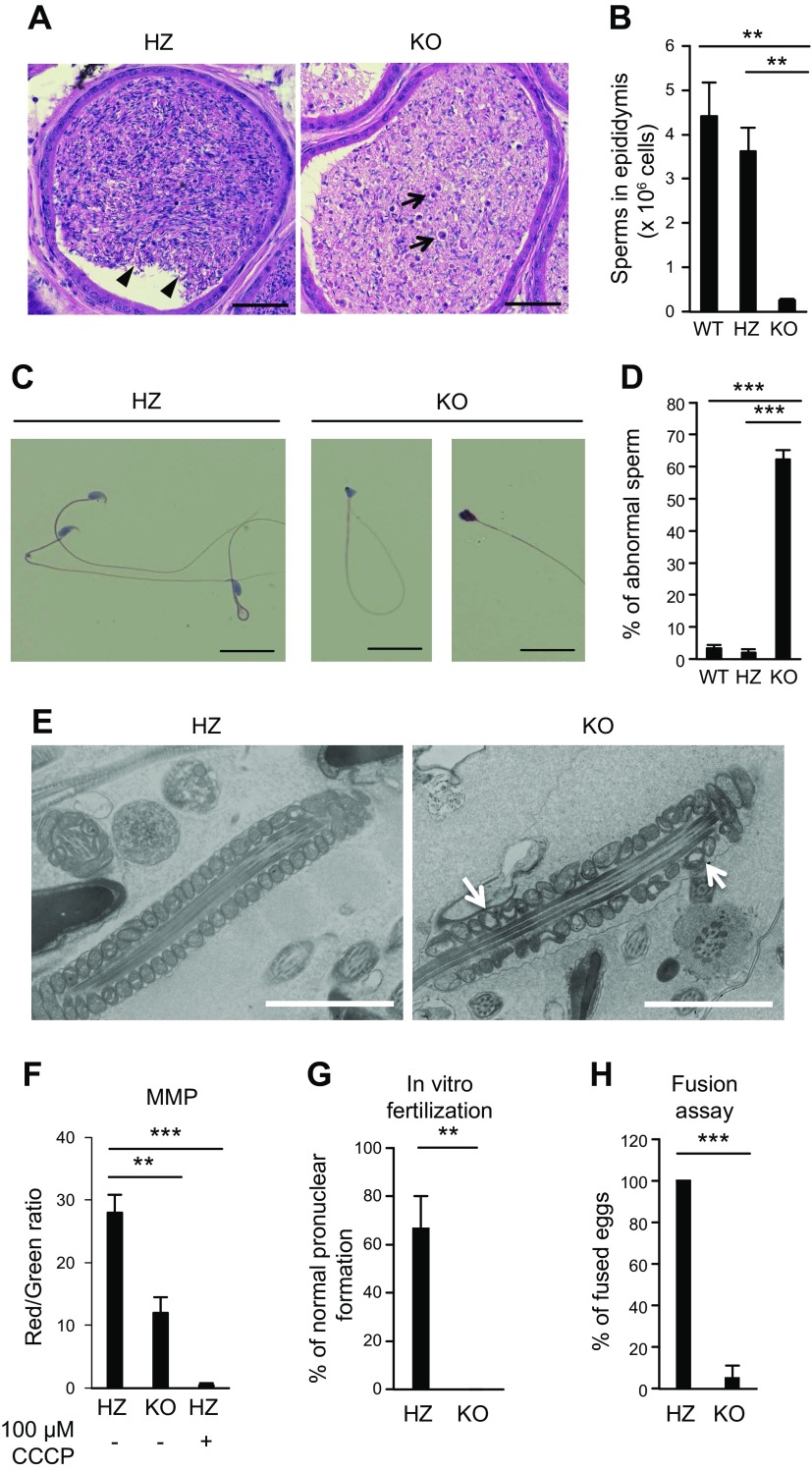
The function and quantity of ACSL6 KO sperm were disordered. *A*) Representative images of HE staining of the ACSL6 HZ and KO cauda epididymis. Arrowheads indicate normal sperm with tails and heads. Arrows point to cell debris for the KO mice. *B*) The numbers of WT, HZ, and KO sperm from the cauda epididymis. Data are presented as means ± sem (*n* = 4). *C*) HE staining of HZ and KO sperm. *D*) Percentage of abnormal sperm from WT, HZ, and KO mice. Data are presented as means ± sem (*n* = 4). *E*) FE-SEM images of ACSL6 HZ and KO epididymis. The arrows show the mitochondria devoid of content. *F*) MMP measured by flow cytometry. *G*, *H*) Successful IVF with (*G*) or without (*H*) the ZP. Scale bars, 2 µm. Data are presented as means ± sem (*n* = 3). **P < 0.01, ***P < 0.001 [Tukey’s honestly significant difference (HSD) test (*B*, *D, F*); unpaired Student’s *t* test (*G*, *H*)].

Sperm is stored in the cauda epididymis. Thus, we examined the number and function of sperm from the cauda epididymis of ACSL6 HZ and KO mice to determine male fertility (28). HE staining revealed that the cauda epididymis of ACSL6 KO mice contained abnormal sperm and cell debris (**Fig. 3*A***). Consistent with the histologic findings, the number of sperm isolated from the cauda epididymis of ACSL6 KO mice was much lower than that of the HZ mice (Fig. 3*B*). A closer examination of the sperm morphology demonstrated that the majority of the ACSL6 KO sperm were abnormally shaped and had irregularly sized heads (Fig. 3*C, D*). MMP was measured by a fluorescent dye, JC-1, and we found that MMP was decreased (−50%) in the KO sperm, as shown in Fig. 3*F*. Also, electron microscopy identified uneven and void mitochondria in the KO sperm, as shown Fig. 3*E*. To examine the fertility of the ACSL6 KO sperm, we performed IVF using WT oocytes. We found that the oocytes could not be fertilized by the KO sperm even though the KO and HZ sperm were used at the same concentration (Fig. 3*G*). To examine whether the ACSL6 KO sperm were unable to fuse with oocytes, we removed the ZP from oocytes prior to IVF. In contrast with the HZ sperm that showed a 100% fusion ratio, only 5% of the KO sperm could fuse with the oocytes, indicating that the fusion capacity of ACSL6 KO sperm was severely defective (Fig. 3*H*). To address the developmental potential of embryos derived from ACSL6 KO sperm, we performed intracytoplasmic sperm injection (ICSI). We assessed the pronuclear formation ratio and the cleavage ratio at 6 and 24 h after ICSI, respectively, and found that the pronuclear formation ratio in oocytes injected with ACSL6 KO sperm was smaller than that of the HZ sperm (**Table 2**). In addition, the cleavage ratio in the KO group was smaller than that of the HZ group (Table 2). These results indicate that not only the fertilization capacity but also the developmental capacity of ACSL6 sperm are diminished, which could account for male infertility observed with ACSL6 KO mice.

**TABLE 2:** Fertilization and cleavage ratios after ICSI for HZ and KO sperm

Genotype	Pronuclear formation (%)	Cleavage (%)
HZ	33/42 (78.6)	32/42 (76.2)
KO	33/67 (49.3)	22/67 (32.8)

### Disordered sperm release and ES apoptosis

**Figure 4:**
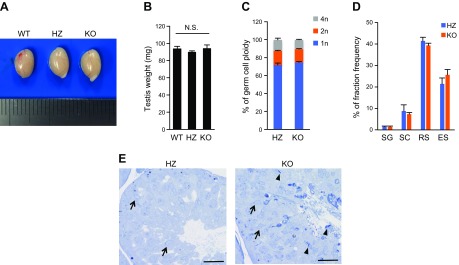
Sperm release was delayed in ACSL6 KO mice. *A*, *B*) The appearance (*A*) and weight (*B*) of testes from WT, HZ, and KO mice. N.S., not significant. Data are presented as means ± sem (*n* = 4). *C*) Germ cell ploidy in HZ and KO mice. Data are presented as means ± sem (*n* = 3). *D*) Fraction frequency of isolated germ cells. Data are presented as means ± sem (*n* = 3). *E*) Toluidine blue staining of stage IX seminiferous tubules from ACSL6 HZ and KO mice. The arrows indicate the start of RS elongation, which is characteristic of stage IX tubules (46). The arrowheads point to the ESs. Scale bars, 25 µm. *B*) Tukey’s honestly significant difference. *C*, *D*) No significant change was detected; unpaired Student’s *t* test.

**Figure 5:**
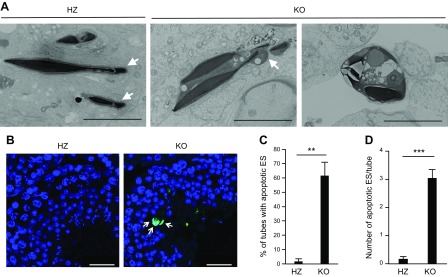
Apoptosis of ESs in ACSL6 KO mice. *A*) FE-SEM images of ACSL6 HZ and KO testes. The arrows show the acrosomes. Scale bars, 4 µm. *B*) TUNEL staining of ACSL6 HZ and KO seminiferous tubules. Nuclei are stained blue (Hoechst). Apoptotic cells are green (TUNEL). The arrows indicate ES undergoing apoptosis. Scale bars, 30 µm. *C*, *D*) The percentage of tubes with apoptotic ES (*C*) and the number of apoptotic ES per tubules (*D*). Data are presented as means ± sem (*n* = 3). **P < 0.01, ***P < 0.001 (unpaired Student’s *t* test).

Abnormal spermatogenesis was attributed to disordered sperm release in the testes of LPAAT3 KO mice (5). To determine the stage of sperm release, we used toluidine blue staining to identify ESs in the ACSL6 KO testis. Although there were no significant changes in testis weight or testicular cell populations, many ESs were observed in the stage IX seminiferous tubules (**Fig. 4**). These results indicate that sperm release was disordered in ACSL6 KO mice, without a change in development or cell differentiation. Field emission scanning electron microscopy (FE-SEM) of stage IX seminiferous tubules revealed abnormally shaped acrosomes in the ESs, which was similar to the phenotype observed in LPAAT3 KO mice (**Fig. 5*A***). However, many vesicular structures containing ESs were uniquely observed in the ACSL6 KO mouse–derived ESs (Fig. 5*A*). Because this structure was reminiscent of ES apoptosis, we performed TUNEL staining (29, 30). Notably, more TUNEL-positive ESs were observed in ACSL6 KO mice compared with HZ mice (Fig. 5*B–D*). In contrast, we found that the serum concentrations of sex hormones (*e.g.*, FSH, LH, and testosterone), which regulate ES cell survival and sperm release (31), were not significantly different between HZ and KO mice (**Table 3**).

**TABLE 3:** Serum sex hormone levels in ACSL6 HZ and KO mice

Hormone	HZ	KO
FSH (ng/ml)	24.34 ± 1.34	23.10 ± 3.19
LH (ng/ml)	5.24 ± 1.08	6.23 ± 1.70
Testosterone (ng/ml)	15.59 ± 0.90	17.0 ± 2.14

Data are presented as means ± sem (*n* = 4). No significant changes were detected; unpaired Student’s *t* test.

## DISCUSSION

In this study, we demonstrated that ACSL6 is involved in the selective enrichment of LCPUFA-containing membrane phospholipids in differentiating spermatids, which supports normal spermatogenesis. Based on human and animal studies, it has been reported that dietary intake of DHA correlates well with increased sperm number and function (32). Conversely, decreased testicular DPA levels are correlated with male infertility (4). DHA and DPA are highly enriched in the testis and spermatozoa in the forms of membrane phospholipids, suggesting those phospholipid species have some important roles in maintaining normal testicular function (4, 32, 33). These data are consistent with the finding that LPAAT3 KO mice have a defect in selective DHA incorporation into phospholipids and also display severe male infertility with abnormal sperm morphology (5). ACSL6 KO and LPAAT3 KO mice have a similar phenotype: decreased sperm fertilization and number and unreleased spermatids. However, remarkably, ES apoptosis was observed in ACSL6 KO but not LPAAT3 KO mice. This difference could stem from the type of decreased LCPUFA-containing phospholipids. In ACSL6 KO mice, DHA- and DPA-containing phospholipids were partially reduced (∼50% for DHA, ∼20% for DPA), whereas DHA- but not DPA-containing phospholipids were dramatically reduced in LPAAT3 KO mice. The precise function of DHA- and DPA-containing phospholipids in spermatogenesis is still poorly understood. Nevertheless, in the previous report on male infertility in LPAAT3 KO mice, it was discussed that membrane flexibility increased by LCPUFA-containing phospholipids may support the endocytosis of excess cytoplasm at the late stage of spermatogenesis (5).

ACSL family enzymes catalyze a wide range of fatty acids to form acyl-CoAs, with some substrate preferences (6, 34–36). Lipidomic profiling of ACSL6 KO mice revealed significant reductions of DHA (22:6)- and DPA (22:5)-containing phospholipids with a compensatory increase in other polyunsaturated fatty acid (PUFA)-containing species, such as AA (20:4), in differentiating spermatids. This type of change is not observed in the KO mice for other ACSL family members (10, 37–41), suggesting that ACSL6 has selectivity for chain length and double bonds in PUFA structures *in vivo*. Indeed, the total ACS activity was significantly reduced for DHA and DPA, whereas no change was observed for palmitate and AA in ACSL6 KO testis. Because *ACSL6* and *LPAAT3* are highly coexpressed in spermatids, there might be some functional coupling of these enzymes to efficiently and selectively incorporate LCPUFA into membrane phospholipids *in vivo*.

Delta-6 fatty acid desaturase KO mice fed an AIN93G diet exhibit spermatogenic arrest between stages RS and ES that is accompanied by decreased LCPUFA levels in the testis (42, 43). In addition, elongation of very-long-chain fatty acids protein 2 KO mice display complete arrest of spermatogenesis with reduced very-long-chain PUFA levels in the testis (44). Ceramide synthase 3 is an enzyme expressed in SCs that generates ultra-long polyunsaturated ceramides; ceramide synthase 3 KO mice display spermatogenic arrest at RS (45). These results clearly indicate the critical role of LCPUFAs in normal spermatogenesis, but further investigation is required to identify which LCPUFA-containing species are important for conferring a spermatoprotective effect *in vivo*.

In summary, we identified ACSL6 as a critical enzyme that enriches LCPUFA-containing phospholipids in the testis and is essential for normal male fertility by regulating proper spermatogenesis.

## Supplementary Material

This article includes supplemental data. Please visit *http://www.fasebj.org* to obtain this information.

## Abbreviations

AA: arachidonic acid
ACSL: long-chain acyl-coenzyme A (CoA) synthetase
CRISPR/Cas9: clustered regularly interspaced short palindromic repeats/CRISPR-associated protein-9 nuclease
DHA: docosahexaenoic acid
DPA: docosapentaenoic acid
ES: elongated spermatid
FE-SEM: field emission scanning electron microscopy
FSH: follicle stimulating hormone
GAPDH: glyceraldehyde 3-phosphate dehydrogenase
HE: hematoxylin and eosin
HZ: heterozygous
ICSI: intracytoplasmic sperm injection
IVF: *in vitro* fertilization
KO: knockout
KSOM: potassium simplex optimized medium
LC: liquid chromatography
LCPUFA: long-chain PUFA
LPAAT3: lysophospholipid acyltransferase 3
MMP: mitochondrial membrane potential
MS: mass spectrometry
PC: phosphatidylcholine
PUFA: polyunsaturated fatty acid
RS: round spermatid
SC: spermatocyte
sgRNA: single guide RNA
UPLC: ultra-performance LC
WT: wild type
ZP: zona pellucida

## References

[1] Spector A. A., Kim H. Y. (2015) Discovery of essential fatty acids. J. Lipid Res. 56, 11–21 25339684 10.1194/jlr.R055095 PMC4274059

[2] Lands B. (2017) Highly unsaturated fatty acids (HUFA) mediate and monitor food’s impact on health. Prostaglandins Other Lipid Mediat. 133, 4–10 28535956 10.1016/j.prostaglandins.2017.05.002

[3] Hishikawa D., Valentine W. J., Iizuka-Hishikawa Y., Shindou H., Shimizu T. (2017) Metabolism and functions of docosahexaenoic acid-containing membrane glycerophospholipids. FEBS Lett. 591, 2730–2744 28833063 10.1002/1873-3468.12825 PMC5639365

[4] Zanetti S. R., Maldonado E. N., Aveldaño M. I. (2007) Doxorubicin affects testicular lipids with long-chain (C18-C22) and very long-chain (C24-C32) polyunsaturated fatty acids. Cancer Res. 67, 6973–6980 17638909 10.1158/0008-5472.CAN-07-0376

[5] Iizuka-Hishikawa Y., Hishikawa D., Sasaki J., Takubo K., Goto M., Nagata K., Nakanishi H., Shindou H., Okamura T., Ito C., Toshimori K., Sasaki T., Shimizu T. (2017) Lysophosphatidic acid acyltransferase 3 tunes the membrane status of germ cells by incorporating docosahexaenoic acid during spermatogenesis. J. Biol. Chem. 292, 12065–12076 28578315 10.1074/jbc.M117.791277 PMC5519358

[6] Mashek D. G., Li L. O., Coleman R. A. (2007) Long-chain acyl-CoA synthetases and fatty acid channeling. Future Lipidol. 2, 465–476 20354580 10.2217/17460875.2.4.465 PMC2846691

[7] Marszalek J. R., Kitidis C., Dirusso C. C., Lodish H. F. (2005) Long-chain acyl-CoA synthetase 6 preferentially promotes DHA metabolism. J. Biol. Chem. 280, 10817–10826 15655248 10.1074/jbc.M411750200

[8] Iijima H., Fujino T., Minekura H., Suzuki H., Kang M. J., Yamamoto T. (1996) Biochemical studies of two rat acyl-CoA synthetases, ACS1 and ACS2. Eur. J. Biochem. 242, 186–190 8973631 10.1111/j.1432-1033.1996.0186r.x

[9] Lee E. J., Kim H. C., Cho Y. Y., Byun S. J., Lim J. M., Ryoo Z. Y. (2005) Alternative promotion of the mouse acyl-CoA synthetase 6 (mAcsl6) gene mediates the expression of multiple transcripts with 5′-end heterogeneity: genetic organization of mAcsl6 variants. Biochem. Biophys. Res. Commun. 327, 84–93 15629433 10.1016/j.bbrc.2004.11.141

[10] Chouinard-Watkins R., Bazinet R. P. (2018) ACSL6 is critical for maintaining brain DHA levels. Proc. Natl. Acad. Sci. USA 115, 12343–12345 30446610 10.1073/pnas.1817557115 PMC6298068

[11] Soupene E., Dinh N. P., Siliakus M., Kuypers F. A. (2010) Activity of the acyl-CoA synthetase ACSL6 isoforms: role of the fatty acid Gate-domains. BMC Biochem. 11, 18 20429931 10.1186/1471-2091-11-18 PMC2868784

[12] Horii T., Arai Y., Yamazaki M., Morita S., Kimura M., Itoh M., Abe Y., Hatada I. (2014) Validation of microinjection methods for generating knockout mice by CRISPR/Cas-mediated genome engineering. Sci. Rep. 4, 4513 24675426 10.1038/srep04513 PMC5380110

[13] Schmittgen T. D., Livak K. J. (2008) Analyzing real-time PCR data by the comparative C(T) method. Nat. Protoc. 3, 1101–1108 18546601 10.1038/nprot.2008.73

[14] Ikeda K. (2015) Mass spectrometric analysis of phospholipids by target discovery approach. In Bioactive Lipid Mediators (Yokomizo T., and Murakami M., eds.), pp. 349–356, Springer, Tokyo, Japan

[15] Aoyagi R., Ikeda K., Isobe Y., Arita M. (2017) Comprehensive analyses of oxidized phospholipids using a measured MS/MS spectra library. J. Lipid Res. 58, 2229–2237 28874441 10.1194/jlr.D077123 PMC5665662

[16] Tsugawa H., Cajka T., Kind T., Ma Y., Higgins B., Ikeda K., Kanazawa M., VanderGheynst J., Fiehn O., Arita M. (2015) MS-DIAL: data-independent MS/MS deconvolution for comprehensive metabolome analysis. Nat. Methods 12, 523–526 25938372 10.1038/nmeth.3393 PMC4449330

[17] Bligh E. G., Dyer W. J. (1959) A rapid method of total lipid extraction and purification. Can. J. Biochem. Physiol. 37, 911–917 13671378 10.1139/o59-099

[18] Fuchs B., Schiller J., Süss R., Schürenberg M., Suckau D. (2007) A direct and simple method of coupling matrix-assisted laser desorption and ionization time-of-flight mass spectrometry (MALDI-TOF MS) to thin-layer chromatography (TLC) for the analysis of phospholipids from egg yolk. Anal. Bioanal. Chem. 389, 827–834 17673987 10.1007/s00216-007-1488-4

[19] Bartlett G. R. (1959) Phosphorus assay in column chromatography. J. Biol. Chem. 234, 466–468 13641241

[20] Narita T., Naganuma T., Sase Y., Kihara A. (2016) Long-chain bases of sphingolipids are transported into cells via the acyl-CoA synthetases. Sci. Rep. 6, 25469 27136724 10.1038/srep25469 PMC4853782

[21] Li Q., Zhang S., Berthiaume J. M., Simons B., Zhang G. F. (2014) Novel approach in LC-MS/MS using MRM to generate a full profile of acyl-CoAs: discovery of acyl-dephospho-CoAs. J. Lipid Res. 55, 592–602 24367045 10.1194/jlr.D045112 PMC3934743

[22] Mays-Hoopes L. L., Bolen J., Riggs A. D., Singer-Sam J. (1995) Preparation of spermatogonia, spermatocytes, and round spermatids for analysis of gene expression using fluorescence-activated cell sorting. Biol. Reprod. 53, 1003–1011 8527502 10.1095/biolreprod53.5.1003

[23] Bastos H., Lassalle B., Chicheportiche A., Riou L., Testart J., Allemand I., Fouchet P. (2005) Flow cytometric characterization of viable meiotic and postmeiotic cells by Hoechst 33342 in mouse spermatogenesis. Cytometry A 65, 40–49 15779065 10.1002/cyto.a.20129

[24] Agnihotri S. K., Agrawal A. K., Hakim B. A., Vishwakarma A. L., Narender T., Sachan R., Sachdev M. (2016) Mitochondrial membrane potential (MMP) regulates sperm motility. In Vitro Cell. Dev. Biol. Anim. 52, 953–960 27338736 10.1007/s11626-016-0061-x

[25] Suh M., Merrells K. J., Dick A., Taylor C. G. (2011) Testes of obese rats are highly responsive to n-3 long-chain fatty acids. Br. J. Nutr. 106, 1005–1012 21486514 10.1017/S0007114511001292

[26] Lin D. S., Connor W. E., Wolf D. P., Neuringer M., Hachey D. L. (1993) Unique lipids of primate spermatozoa: desmosterol and docosahexaenoic acid. J. Lipid Res. 34, 491–499 8468532

[27] Griswold M. D. (2016) Spermatogenesis: the commitment to meiosis. Physiol. Rev. 96, 1–17 26537427 10.1152/physrev.00013.2015 PMC4698398

[28] Kumar N., Singh A. K. (2015) Trends of male factor infertility, an important cause of infertility: a review of literature. J. Hum. Reprod. Sci. 8, 191–196 26752853 10.4103/0974-1208.170370 PMC4691969

[29] Chen Y., Wang H., Qi N., Wu H., Xiong W., Ma J., Lu Q., Han D. (2009) Functions of TAM RTKs in regulating spermatogenesis and male fertility in mice. Reproduction 138, 655–666 19602523 10.1530/REP-09-0101

[30] Rainey M. A., George M., Ying G., Akakura R., Burgess D. J., Siefker E., Bargar T., Doglio L., Crawford S. E., Todd G. L., Govindarajan V., Hess R. A., Band V., Naramura M., Band H. (2010) The endocytic recycling regulator EHD1 is essential for spermatogenesis and male fertility in mice. BMC Dev. Biol. 10, 37 20359371 10.1186/1471-213X-10-37 PMC2856533

[31] Ruwanpura S. M., McLachlan R. I., Meachem S. J. (2010) Hormonal regulation of male germ cell development. J. Endocrinol. 205, 117–131 20144980 10.1677/JOE-10-0025

[32] Esmaeili V., Shahverdi A. H., Moghadasian M. H., Alizadeh A. R. (2015) Dietary fatty acids affect semen quality: a review. Andrology 3, 450–461 25951427 10.1111/andr.12024

[33] Lenzi A., Picardo M., Gandini L., Dondero F. (1996) Lipids of the sperm plasma membrane: from polyunsaturated fatty acids considered as markers of sperm function to possible scavenger therapy. Hum. Reprod. Update 2, 246–256 9079417 10.1093/humupd/2.3.246

[34] Fujino T., Kang M. J., Suzuki H., Iijima H., Yamamoto T. (1996) Molecular characterization and expression of rat acyl-CoA synthetase 3. J. Biol. Chem. 271, 16748–16752 8663269 10.1074/jbc.271.28.16748

[35] Oikawa E., Iijima H., Suzuki T., Sasano H., Sato H., Kamataki A., Nagura H., Kang M. J., Fujino T., Suzuki H., Yamamoto T. T. (1998) A novel acyl-CoA synthetase, ACS5, expressed in intestinal epithelial cells and proliferating preadipocytes. J. Biochem. 124, 679–685 9722683 10.1093/oxfordjournals.jbchem.a022165

[36] Kang M. J., Fujino T., Sasano H., Minekura H., Yabuki N., Nagura H., Iijima H., Yamamoto T. T. (1997) A novel arachidonate-preferring acyl-CoA synthetase is present in steroidogenic cells of the rat adrenal, ovary, and testis. Proc. Natl. Acad. Sci. USA 94, 2880–2884 9096315 10.1073/pnas.94.7.2880 PMC20291

[37] Li L. O., Grevengoed T. J., Paul D. S., Ilkayeva O., Koves T. R., Pascual F., Newgard C. B., Muoio D. M., Coleman R. A. (2015) Compartmentalized acyl-CoA metabolism in skeletal muscle regulates systemic glucose homeostasis. Diabetes 64, 23–35 25071025 10.2337/db13-1070 PMC4274800

[38] Killion E. A., Reeves A. R., El Azzouny M. A., Yan Q. W., Surujon D., Griffin J. D., Bowman T. A., Wang C., Matthan N. R., Klett E. L., Kong D., Newman J. W., Han X., Lee M. J., Coleman R. A., Greenberg A. S. (2018) A role for long-chain acyl-CoA synthetase-4 (ACSL4) in diet-induced phospholipid remodeling and obesity-associated adipocyte dysfunction. Mol. Metab. 9, 43–56 29398618 10.1016/j.molmet.2018.01.012 PMC5870107

[39] Meller N., Morgan M. E., Wong W. P., Altemus J. B., Sehayek E. (2013) Targeting of Acyl-CoA synthetase 5 decreases jejunal fatty acid activation with no effect on dietary long-chain fatty acid absorption. Lipids Health Dis. 12, 88 23767941 10.1186/1476-511X-12-88 PMC3699395

[40] Ellis J. M., Li L. O., Wu P. C., Koves T. R., Ilkayeva O., Stevens R. D., Watkins S. M., Muoio D. M., Coleman R. A. (2010) Adipose acyl-CoA synthetase-1 directs fatty acids toward beta-oxidation and is required for cold thermogenesis. Cell Metab. 12, 53–64 20620995 10.1016/j.cmet.2010.05.012 PMC2910420

[41] Ellis J. M., Mentock S. M., Depetrillo M. A., Koves T. R., Sen S., Watkins S. M., Muoio D. M., Cline G. W., Taegtmeyer H., Shulman G. I., Willis M. S., Coleman R. A. (2011) Mouse cardiac acyl coenzyme a synthetase 1 deficiency impairs fatty acid oxidation and induces cardiac hypertrophy. Mol. Cell. Biol. 31, 1252–1262 21245374 10.1128/MCB.01085-10 PMC3067914

[42] Roqueta-Rivera M., Stroud C. K., Haschek W. M., Akare S. J., Segre M., Brush R. S., Agbaga M. P., Anderson R. E., Hess R. A., Nakamura M. T. (2010) Docosahexaenoic acid supplementation fully restores fertility and spermatogenesis in male delta-6 desaturase-null mice. J. Lipid Res. 51, 360–367 19690334 10.1194/jlr.M001180 PMC2803238

[43] Roqueta-Rivera M., Abbott T. L., Sivaguru M., Hess R. A., Nakamura M. T. (2011) Deficiency in the omega-3 fatty acid pathway results in failure of acrosome biogenesis in mice. Biol. Reprod. 85, 721–732 21653892 10.1095/biolreprod.110.089524

[44] Zadravec D., Tvrdik P., Guillou H., Haslam R., Kobayashi T., Napier J. A., Capecchi M. R., Jacobsson A. (2011) ELOVL2 controls the level of n-6 28:5 and 30:5 fatty acids in testis, a prerequisite for male fertility and sperm maturation in mice. J. Lipid Res. 52, 245–255 21106902 10.1194/jlr.M011346 PMC3023544

[45] Rabionet M., Bayerle A., Jennemann R., Heid H., Fuchser J., Marsching C., Porubsky S., Bolenz C., Guillou F., Gröne H. J., Gorgas K., Sandhoff R. (2015) Male meiotic cytokinesis requires ceramide synthase 3-dependent sphingolipids with unique membrane anchors. Hum. Mol. Genet. 24, 4792–4808 26045466 10.1093/hmg/ddv204

[46] Russell D. L., Ettlin A. R., Sinha P. A., Clegg D. E. (1993) Histological and histopathological evaluation of the testis. Int. J. Androl. 16, 83

