# Supplementary material for: Acyl-CoA synthetase 6 regulates long-chain polyunsaturated fatty acid composition of membrane phospholipids in spermatids and supports normal spermatogenic processes in mice
Source: FASEB J. 2019 Nov 25;33(12):14194–203. doi: 10.1096/fj.201901074R (PMC6894091; doi:10.1096/fj.201901074R)
Supplement: Supplementary file 1 [file fj.201901074R.sd1.pdf]

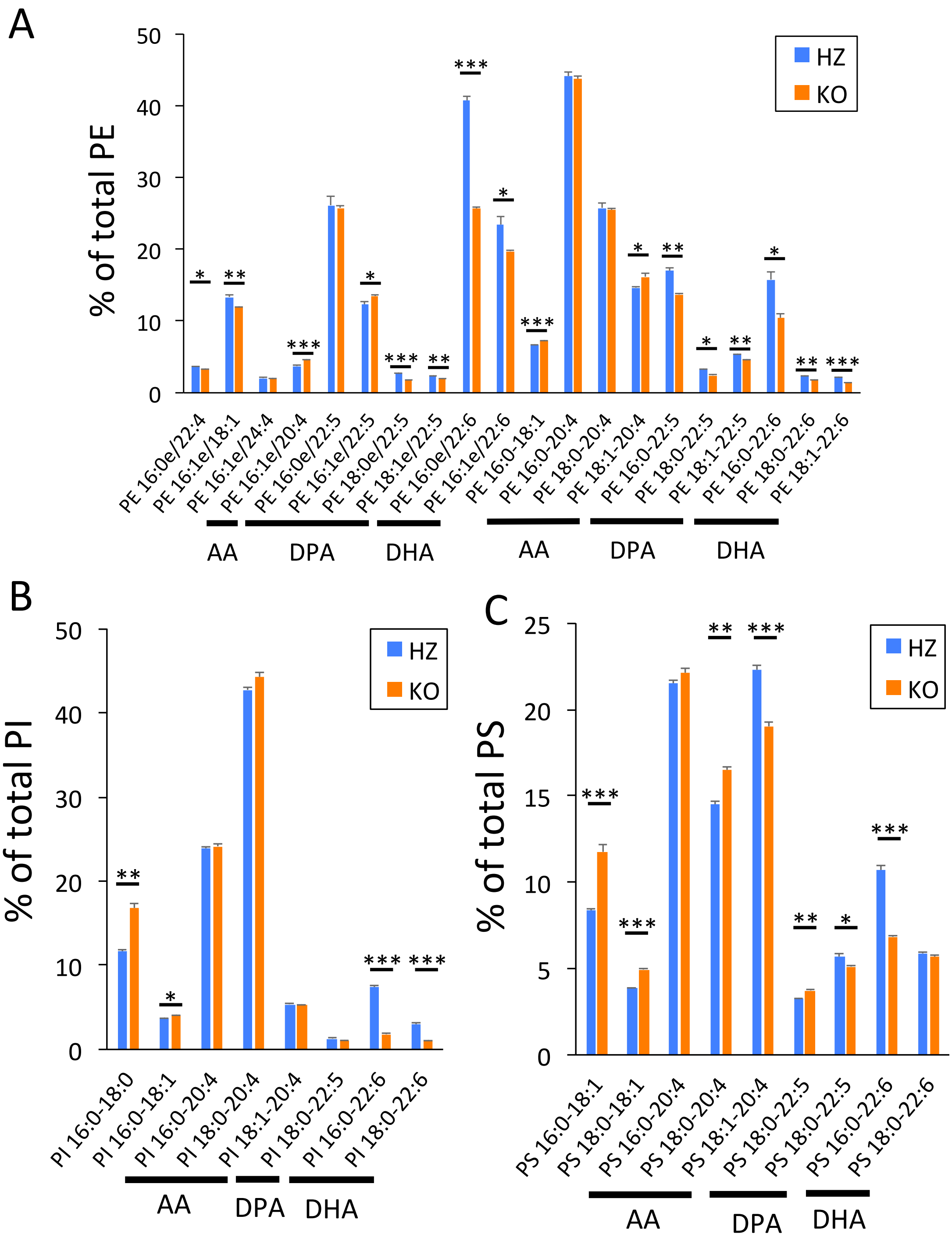

Composition of phospholipid in testis
A-C) percentage of total phosphatidylethanolamine (PE), phosphatidylinositol (PI) and phosphatidylserine (PS). PEs only in excess of 2% are shown. PIs and PSs in excess of 1% are presented. Data are presented as mean ± SEM (n = 3). *p < 0.05, **p < 0.01, *** p < 0.001; unpaired student t test.

Supplementary Fig. S1

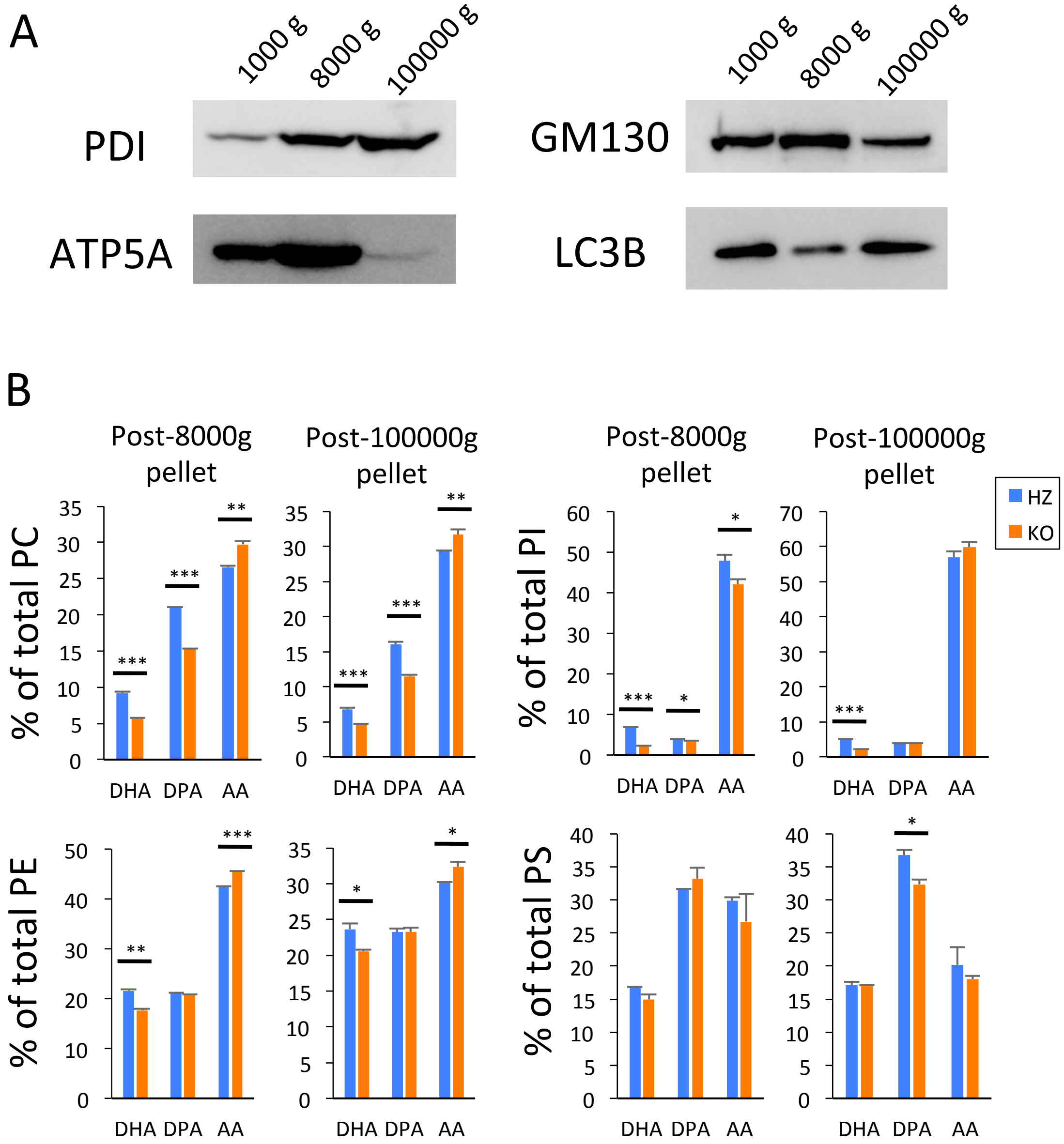

Subcellular fractionation and lipidomic analysis of the testes

A) Representative subcellular fractionation. Pellets of post-1000g, post-8000g and post-100000g centrifugation were analyzed with western blot for PDI (ER marker), ATP5A (mitochondria marker), GM130 (golgi marker) and LC3B (lysosome marker) respectively. *B*) Lipidomic analysis of post-8000g and post-100000g pellets. Data are presented as mean ± SEM (n = 3). *$P < 0.01$, **$P < 0.01$, ***$P < 0.001$; unpaired student t-test.

Supplementary Fig. S2

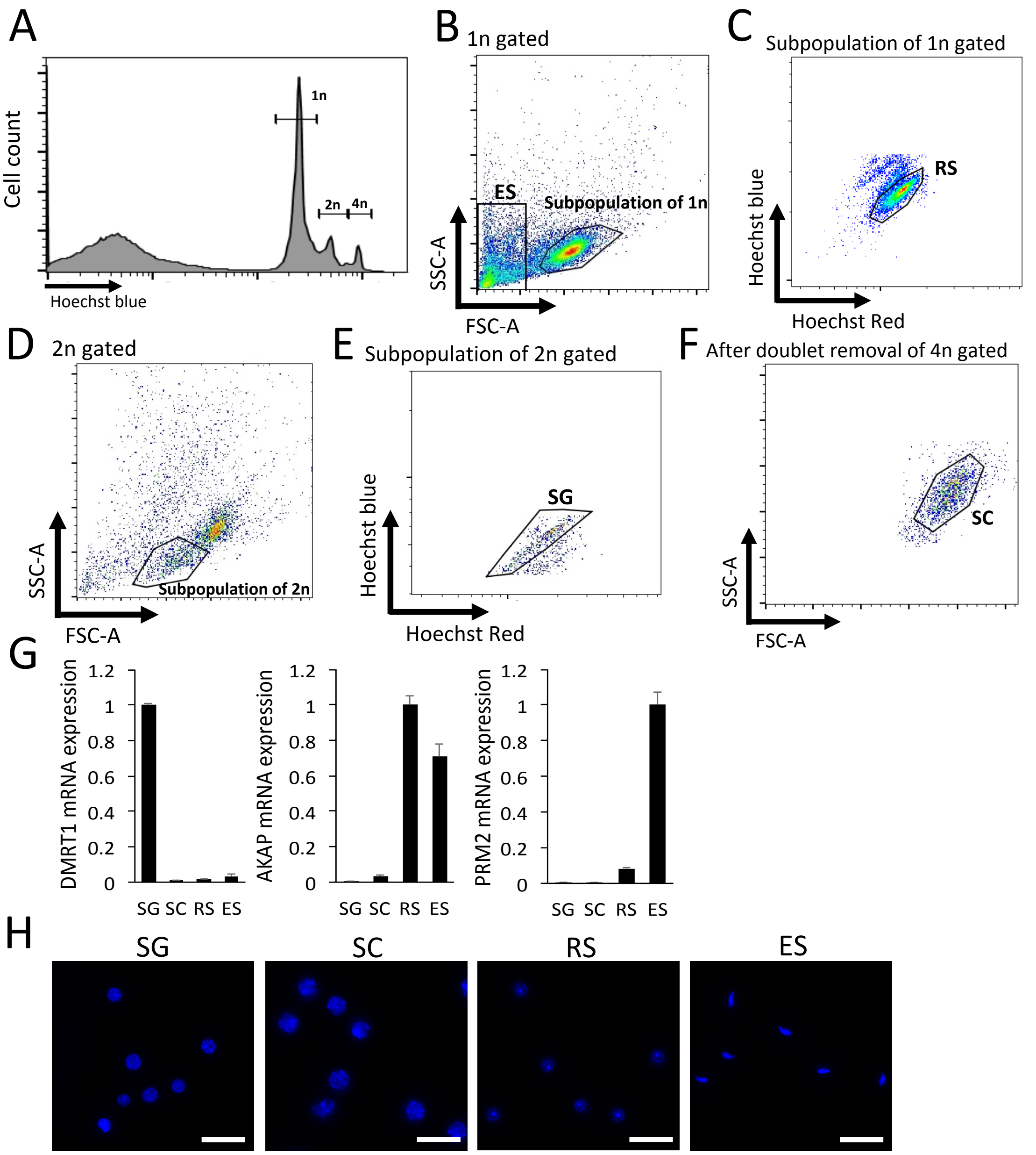

FACS sorting of spermatids

A-F) representative sorting of testicular single cells. A, after dead cell removal using propidium iodide, living cells were classified into three populations based on Hoechst 33342 histogram: haploid (1n), diploid (2n), and tetraploid (4n). B-E, haploid cells and diploid cells were gated with forward scattering and side scattering (*B*, *D*), followed by further purification using Hoechst blue and red respectively (*C*, *E*). F, after doublet cell removal using forward scattering and side scattering, single population of spermatocyte was obtained. spermatogonia (SG), spermatocyte (SC), round spermatid (RS) and elongated spermatid (ES).

G, *H*) successful purification was confirmed with specific gene expression and nuclear shape in each fraction. Gene expressions of differentiation markers in isolated SG, SC, RS and ES (*G*). We used expression markers as follows; doublesex and mab-3 related transcription factor 1 (DMRT1), expressed in spermatogonia; A kinase anchoring protein 3 (AKAP3), expressed in haploid spermatids; protamine 2 (PRM2), predominantly expressed in late spermatids. Data are presented as mean ± SEM (n = 3). *H*) nuclear shapes of isolated spermatids.

Supplementary Fig. S3
